# 3D-printed equipment to decouple (powder) X-ray diffraction sample preparation and measurement

**DOI:** 10.1107/S160057672200293X

**Published:** 2022-04-29

**Authors:** Friederike Fuß, Max Rieckert, Simon Steinhauer, Moritz Liesegang, Günther Thiele

**Affiliations:** a Fachbereich Biologie Chemie Pharmazie, Freie Universität Berlin, Fabeckstraße 34–36, Berlin, 14195, Germany; b Institut für Geologische Wissenschaften, Mineralogie–Petrologie, Freie Universität Berlin, Malteserstraße 74–100, Berlin, 12249, Germany

**Keywords:** 3D printing, cryo-storage, single crystals, powders, diffraction, home sources

## Abstract

An alternative storage method to separate sample preparation from single-crystal and powder X-ray diffraction measurements at home source diffractometers is described. For single crystals, a setup is presented which allows storage of preselected crystals under cryogenic and ambient temperatures. For powders, a disposable sample holder is introduced. The method is suitable for the storage of air- and moisture-sensitive samples. Equipment made of biodegradable polylactic acid is produced by 3D printing and can be adapted to individual needs. As 3D printers are widely available at research institutions nowadays, models of the presented equipment are provided for the reader to allow easy reproduction.

## Abstract 2

A method to separate sample preparation from X-ray diffraction and powder X-ray diffraction measurements is presented. The necessary equipment is accessible at low cost by 3D printing.

## 1. Introduction

High-sensitivity detectors combined with narrowly focused X-rays reduce the time for unit-cell determination of small-molecule single crystals in X-ray diffraction (XRD) experiments with home source diffractometers to 5 min or less (Ott & Stuerzer, 2016 ▸). Selecting and mounting a suitable crystal can take longer than this. The storage of mounted crystals enables a more efficient use of expensive and often restricted measurement time.

In macromolecular and biochemical crystallography, the storage of mounted crystals in liquid nitro­gen is an established method. These systems are automated and frequently installed at synchrotron beamlines (Garman, 2014 ▸). The setup often includes a cylindrical block called a puck to store the pins with mounted crystals. Labelled pucks for sample assignment can be stacked and submerged in tanks with liquid nitro­gen. Storage systems with pucks for automated measurement setups are commercially available and can be also used at home sources. There are different types of pucks on the market, most made of metal. The different designs either enclose the pins completely or include additional covers to ensure continuous submersion in liquid nitro­gen.

Improved pins and pucks to increase the number of pins fitting into one puck have been developed for automated setups to provide higher space efficiency during storage (Papp *et al.*, 2017 ▸). The acquisition and maintenance of automated measurement setups is usually not economically feasible for universities, where the number of (in)organic small-molecule samples does not reach the capacity of automated setups. At the same time, access to shared synchrotron sources is limited and does not fit the need to measure samples occasionally. Operating a diffractometer manually allows for assessing the crystal’s quality before starting a measurement. If the crystal is weakly diffracting or of poor quality, replacing it with pre-selected, mounted and stored crystals accelerates the overall process significantly. The existing storage solutions made for automated setups ease the work with home sources but need a considerable investment to acquire a starting kit.

Samples for powder X-ray diffraction analysis are often prepared on silicon single-crystal discs, as these cause only negligible background noise in the data set. These discs are multi-use and can be reused several hundred times, if not attacked by chemicals or broken by physical impacts. Silicon is not chemically stable against all research samples and replace­ment of these sample holders is expensive. Air-sensitive samples can also be prepared via this technique but will need to be handled in a glovebox before the measurement.

3D printers are widely commercially available, starting at a few hundred euros for a simple fused deposition modelling (FDM) device. A 3D printer is available in many laboratories and institutes nowadays. These allow fast manufacturing of individually tailored reactionware and specific equipment for analytical applications created at a low cost (Kitson *et al.*, 2014 ▸). Examples for applications in diffraction experiments and X-ray applications range from reactionware, to moulds for supplementary equipment and even to loops for crystal mounting. Achilli *et al.* (2016 ▸) designed and tested 3D-printed electrochemical cells to study the mechanism of photoelectrochemical water splitting by X-ray absorption spectroscopy. Printing with a filament made from boron carbide mixed with a polymer enables the fabrication of boron carbide apertures in small-angle neutron scattering experiments (Olsson & Rennie, 2016 ▸). Miller *et al.* (2011 ▸) used 3D-printed models to make apertures for X- and γ-rays in single-photon emission computed tomography applications by cold casting (pouring a mixture of metal powder and resin into a mould). The existing technique of *in meso in situ* crystallographic characterization of proteins to determine their conformation within a liquid medium was recently improved by 3D-printed sample holders (Huang *et al.*, 2020 ▸). Loops for crystal mounting can be 3D-printed *via* digital light projection stereolithography. The 3D-printed loops show data acquisition properties comparable to commercially available mounts and reusability at a price of less than a cent of material cost per mount (Macdonald *et al.*, 2017 ▸).

Sample storage techniques for non-automated home source diffractometers are not used as frequently as in macromolecular crystallography. Here we introduce two methods for separating the process of crystal selection and mounting, as well as the preparation of powder samples, from the actual X-ray diffraction experiment using 3D-printed equipment made from polylactic acid (PLA).

## 2. An alternative method for the separation of sample preparation from the XRD measurement of single crystals using 3D-printed equipment

In the following, 3D-printed holders to store mounted small-molecule single crystals are presented. With the associated sample preparation procedure, air- and moisture-stable crystals can be stored in a dust-free environment, while sensitive samples are stored in liquid nitro­gen to prevent decomposition. The design is quite similar to some commercial solutions but provides an alternative, customizable approach. Where a 3D printer is already available, it allows researchers to try cryogenic sample storage for home source diffractometers before investing in cost-intensive commercial solutions. If proven useful, the change to a commercial solution might still be convenient. To illustrate the method, the entire process, from isolating single crystals to starting the measurement, will be described, including the steps of crystal selection at the microscope, storage and measurement setup. A brief video of the described procedure can be found in the supporting information (SI).

The selection of crystals under the microscope is performed as usual in oil to preserve crystals from contact with moisture and air and make them stick to the pin. To prevent any crystals from sliding off the pin, crystallographic oil that hardens at liquid-nitro­gen temperatures should be applied. For air-stable samples stored at room temperature, oil that is sufficiently viscous to prevent the crystals from sliding off the pin should be chosen.

**Figure 1:**
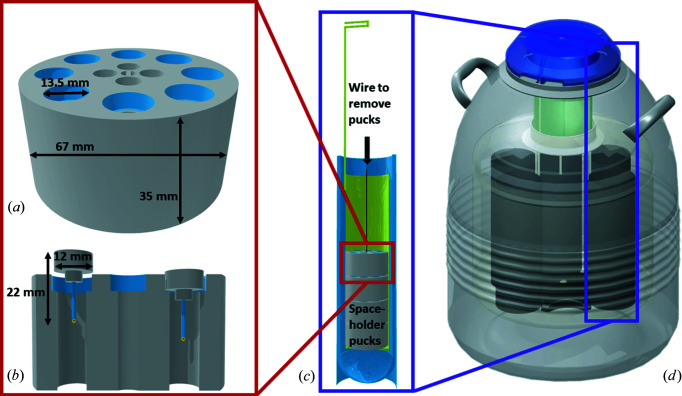
Schematic depictions of (*a*) a 3D-printed puck, (*b*) a cross section of the puck containing commercially available pins, (*c*) a cross section of a sieve (in green) with stacked pucks in a standard laboratory dewar during crystal selection and (*d*) sieves in a cryogenic sample storage dewar. Image of schematic storage dewar taken from IVF Store (2021 ▸).

Several commercially available pins with magnetic bases are needed. Selected crystals are mounted on the pins and stored upside down in 3D-printed holders (pucks), which are submerged in liquid nitro­gen [Figs. 1 ▸(*a*) and 1 ▸(*b*)]. Up to eight pins can be stored on individually labelled positions within one of the pucks presented here. In case a sample is not single crystalline or of sufficient quality, several potentially suitable crystals should be selected and stored, from which the best one can be chosen after evaluation at the diffractometer. Multiple distinctively labelled pucks are threaded onto a wire and located inside the sample holder, the ‘sieve’, of a cryogenic sample storage dewar and immersed in liquid nitro­gen [Fig. 1 ▸(*c*)]. The wire that aligns the pucks and allows a careful introduction to and removal from the sieve should end at the upper rim of the sieve to avoid entanglement of the wires within the sample storage dewar. When the sample selection is finished, the labelled (*e.g.* colour-coded) sieve is transferred into the sample storage dewar, filled with dry liquid nitro­gen, where the samples are kept until measurement time is available [Fig. 1 ▸(*d*)]. With the labelled position of the pin within the puck and the labels of the puck and the sieve, each crystal can be identified unambiguously.

**Figure 2:**
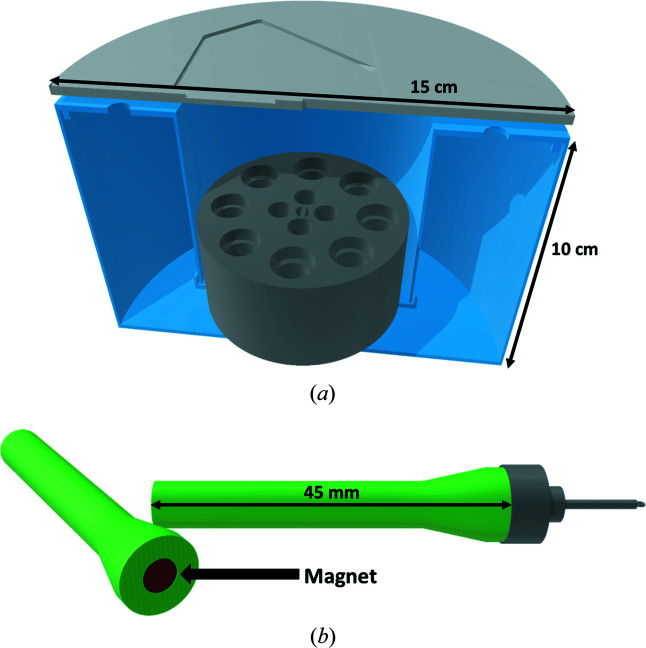
Schematic depictions of (*a*) a 3D-printed puck in a cross section of a 3D-printed dewar and lid and (*b*) a 3D-printed magnetic wand to help transfer the pins from the puck to the diffractometer.

For the measurement, a sieve with multiple pucks is removed from the sample storage dewar and transferred into a dewar filled with liquid nitro­gen. The upper puck is removed, *e.g*. with common crucible tongs, and put into a custom-made 3D-printed dewar, enabling easy access of the pins [Fig. 2 ▸(*a*)]. The sieve with further pucks is transferred back into the sample storage dewar. The 3D-printed dewar is filled with polyurethane (PU) foam for insulation prior to the first usage. Using a 3D-printed customized magnetic wand [Fig. 2 ▸(*b*)], a pin tong or a cryo tong, the pins with the pre-selected crystals can be mounted onto the diffractometer for cell determination and, if suitable, a full measurement.

Exposure of the cooled crystals to moist air can cause the formation of ice on their surface. This can produce erroneous diffraction patterns including reflections from ice crystals, while thawing the crystals to remove ice can lead to the decomposition of crystals coming into contact with the meltwater. The PU-foam filling of the 3D-printed dewar provides less thermal insulation than an evacuated glass dewar. This leads to a higher evaporation rate of liquid nitro­gen in the 3D-printed dewar, which additionally protects the crystals from crystallizing moisture. Sample transfer to the goniometer and subsequent cell determination can therefore be done without haste as long as sufficient liquid nitro­gen is in the dewar.

The transfer from the puck to the goniometer should be done in one smooth and swift motion to prevent the formation of ice on the crystal during mounting. A pin tong, a cryo tong or a customized 3D-printed magnetic wand can be used for this. At some home sources, the use of a pin tong makes it necessary to remove the collimator to mount a pin. In this case, a customized magnetic wand of smaller size is advantageous. With the help of the magnetic wand, the crystal can be positioned in the protective gas flow of the diffractometer. The magnetic base can then be transferred from the magnetic wand onto the goniometer.

Storage in liquid nitro­gen means that the magnetic base is very cold, and protective gloves with sufficient flexibility are needed. We have found thin cotton gloves to be a suitable combination of temperature protection and handling precision.

Over time, moisture crystallizes in liquid nitro­gen, leading to the formation of ice. The ice tends to accumulate at the bottom of the dewar, close to or even on the stored crystals. This can lead to erroneous diffraction patterns, too. The formation of ice can be reduced by limiting the exposure of the liquid nitro­gen to air at every step of the procedure. Covering the dewars with 3D-printed lids, polystyrene or aluminium foil during the transfer of the pins to the pucks, during crystal selection and during the transfer of the pins onto the goniometer is sufficient. The time the sieve is kept in a dewar with liquid nitro­gen at the microscope should be limited to a maximum of two hours. After this time, the sieve should be transferred to the storage dewar or the liquid nitro­gen should be exchanged. Once a measurement is running, the sieve with the remaining samples should be directly returned into the storage dewar after the ice has been removed from the exposed surfaces of the sieve, *e.g.* the handle sticking out of the laboratory dewar.

The addition of two empty ‘space-holder’ pucks at the bottom of the sieve can help to protect the samples from coming into contact with any ice that accumulates at the bottom of the storage dewar [Fig. 1 ▸(*c*)]. Without the use of space-holder pucks, the nitro­gen in the cryogenic sample storage dewar will need to be replaced by fresh and ice-free liquid nitro­gen at regular intervals. Depending on the size of the sample storage dewar, we found intervals of four to eight weeks suitable for capacities of 10.5 and 34.8 l liquid nitro­gen, respectively. Independent of the usage of space-holder pucks, we recommend keeping this interval as a precaution, when the ‘wet’ liquid nitro­gen can be used in the laboratory for other purposes.

The combination of adding the space-holder pucks, limiting the exposure time of liquid nitro­gen to air and regular changes of liquid nitro­gen has proven useful in reducing the number of ice-containing samples. Before these measures were taken, approximately 50% of 800 measured samples in the lowest two pucks of a stack contained additional crystallites of ice. After their implementation, the number of ice-containing samples was reduced to <1% out of 1750. The remaining samples with ice could be attributed to excessive exposure to moist air, *e.g.* during sample selection. Although the level of liquid nitro­gen in the sample storage dewar needs to be carefully monitored at all times, an atmosphere of gaseous nitro­gen will continue to protect the samples from oxygen- and moisture-induced decomposition if the level sinks below the pucks.

### 2.1. Data collection of the stored single crystals

**Figure 3:**
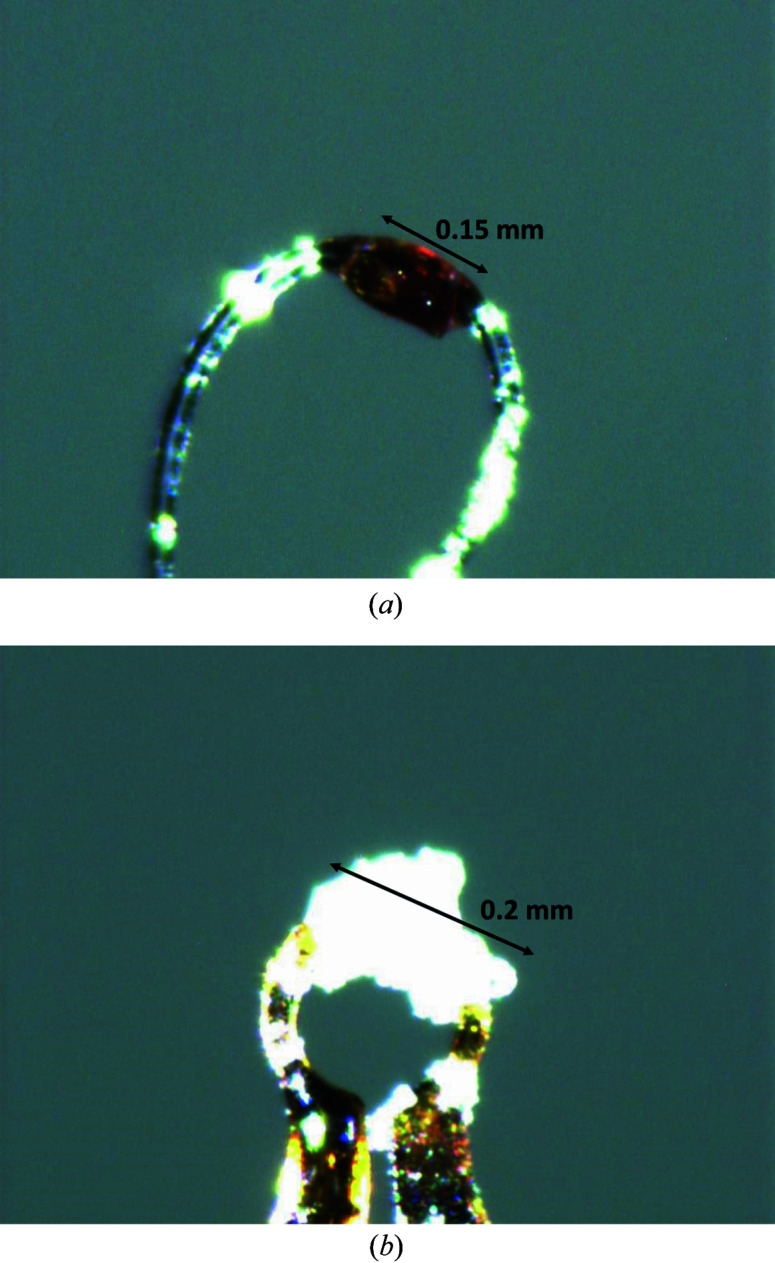
Photographs of mounted crystal. (*a*) An intact Na_4_[FeO_3_] crystal after storage in dry liquid nitro­gen for six days. (*b*) A crystal covered with ice after exposure to air or wet liquid nitro­gen.

The method for storing single crystals reported here has proven suitable for air- and moisture-sensitive compounds. Fig. 3 ▸(*a*) shows a red crystal of Na_4_[FeO_3_] after six days of storage in a 3D-printed puck in the cryogenic storage dewar with the described technique in dry liquid nitro­gen. When exposed to air, the crystal turns colourless and decomposes within 30 s, despite a protective layer of Paraton oil. As a result of exposure of the cooled crystal to moist air and wet liquid nitro­gen, ice can form directly on the sample [Fig. 3 ▸(*b*)]. The obtained modification in all cases was found to be ice I_h_ (*P*6_3_/*mmc*, *a* = 4.506, *c* = 7.346, ICSD-64776; Goto *et al.*, 1990 ▸). In contrast to Na_4_[FeO_3_], the highly reactive sodide [K(C222)]Na (C222 is 4,7,13,16,21,24-hexaoxa-1,10-di­aza­bicyclo­[8.8.8]hexa­cosane; van Eck *et al.*, 1982 ▸) was found to be decomposed after six days of storage. The sodide is extremely sensitive to moisture and air, and decomposes within a few seconds under ambient conditions. The cause of decomposition could not be attributed unambigously to the storage or the exposure to moist air in the different transfer steps. Consequently, very reactive or very temperature sensitive samples need to be stored in closed-bottom pucks to maintain cryogenic conditions. A special case is samples that undergo temperature-induced phase transitions, which might cause crystal fracturing or twinning. These are not suitable for storage and measurement at cryogenic conditions. Therefore air-stable samples found to undergo temperature-induced phase transitions at liquid nitro­gen temperatures can be stored in a dust-free place. Air- and temperature-sensitive samples can be inserted into a puck for a single pin, which is placed in an argon-flushed Schlenk tube at room temperature (Fig. S3).

## 3. A new method for the separation of sample preparation from a powder XRD experiment

Here we introduce 3D-printed PLA sample holders for powder X-ray diffraction (PXRD) measurements. The sample holders are easily produced in a cost-efficient manner and facilitate a rapid measurement setup with high capacities as no sample preparation at the diffractometer is necessary. The sample holders are covered with Scotch Magic Tape to protect sensitive samples from air and moisture before and during the measurement. The method offers a strong alternative to the sample preparation on silicon single-crystal discs for powders that are reactive towards silicon.

**Figure 4:**
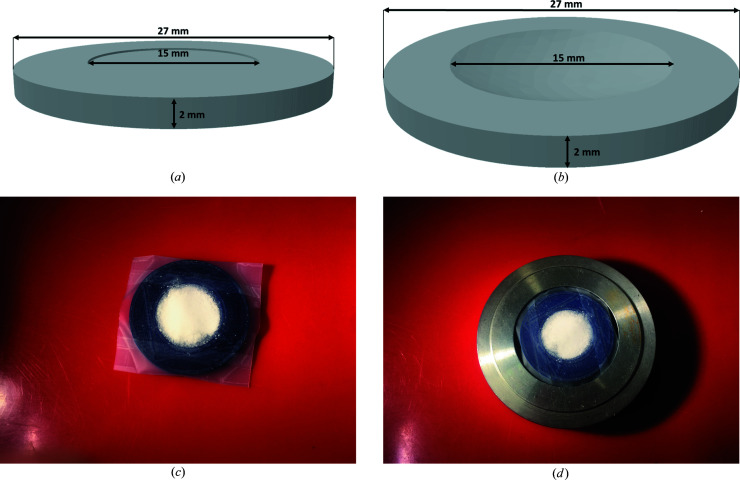
Schematic depictions of (*a*) a 3D-printed sample holder with a shallow depression for very small amounts of powder and (*b*) a sample holder with an indentation for larger amounts of sample material. Photographs of (*c*) a sample holder with sample and covered with Scotch Magic Tape and (*d*) a sample loaded in the sample-holder ring of the diffractometer.

Two different types of 3D-printed sample holders have proven useful [Figs. 4 ▸(*a*) and 4 ▸(*b*)]. A sample holder with a shallow depression is preferred for particularly small amounts of powder. Sample holders with a larger indentation allow an increased thickness of the powder layer.

To prevent displacement of the substance during transport and contact with air, samples are prepared on the 3D-printed sample holders and covered with Scotch Magic Tape [Fig. 4 ▸(*c*)]. The powder sample should be covered with a single flat layer of tape to keep intensities high and avoid dispersion of the solid onto the edge of the sample holder. This particular tape was chosen as it is almost X-ray transparent and amorphous. Air- and moisture-sensitive samples are prepared inside a glovebox where they are stored until measurement time is available. For the subsequent transfer to the diffractometer the samples can be inserted into sealed secondary containers to facilitate an argon atmosphere as long as possible. The as-prepared powder samples can be inserted into device-specific sample holder rings at the diffractometer [Fig. 4 ▸(*d*)]. Automatic sample-changer setups can be used to measure several samples in a row. The low cost of material and production allows a single use. Waste created upon cleaning is minimized by the disposal of the sample holders after the measurement.

### 3.1. Data analysis of powders prepared on the 3D-printed sample holders

**Figure 5:**
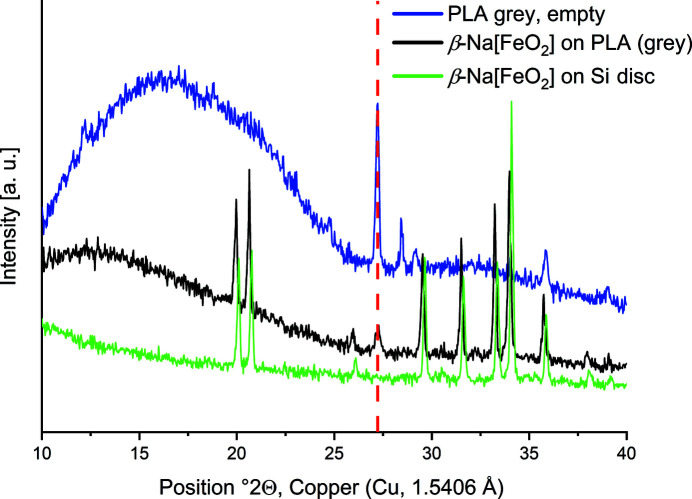
Comparison of PXRD patterns of an empty PLA sample holder (blue) and β-Na[FeO_2_] measured on a PLA sample holder (black) and on a silicon single-crystal disc (green). The red dashed line highlights the most intense PLA reflection, which is also visible in the black diffractogram. Diffractograms were measured with Cu *K*α radiation (1.5406 Å) at 293 K.

**Figure 6:**
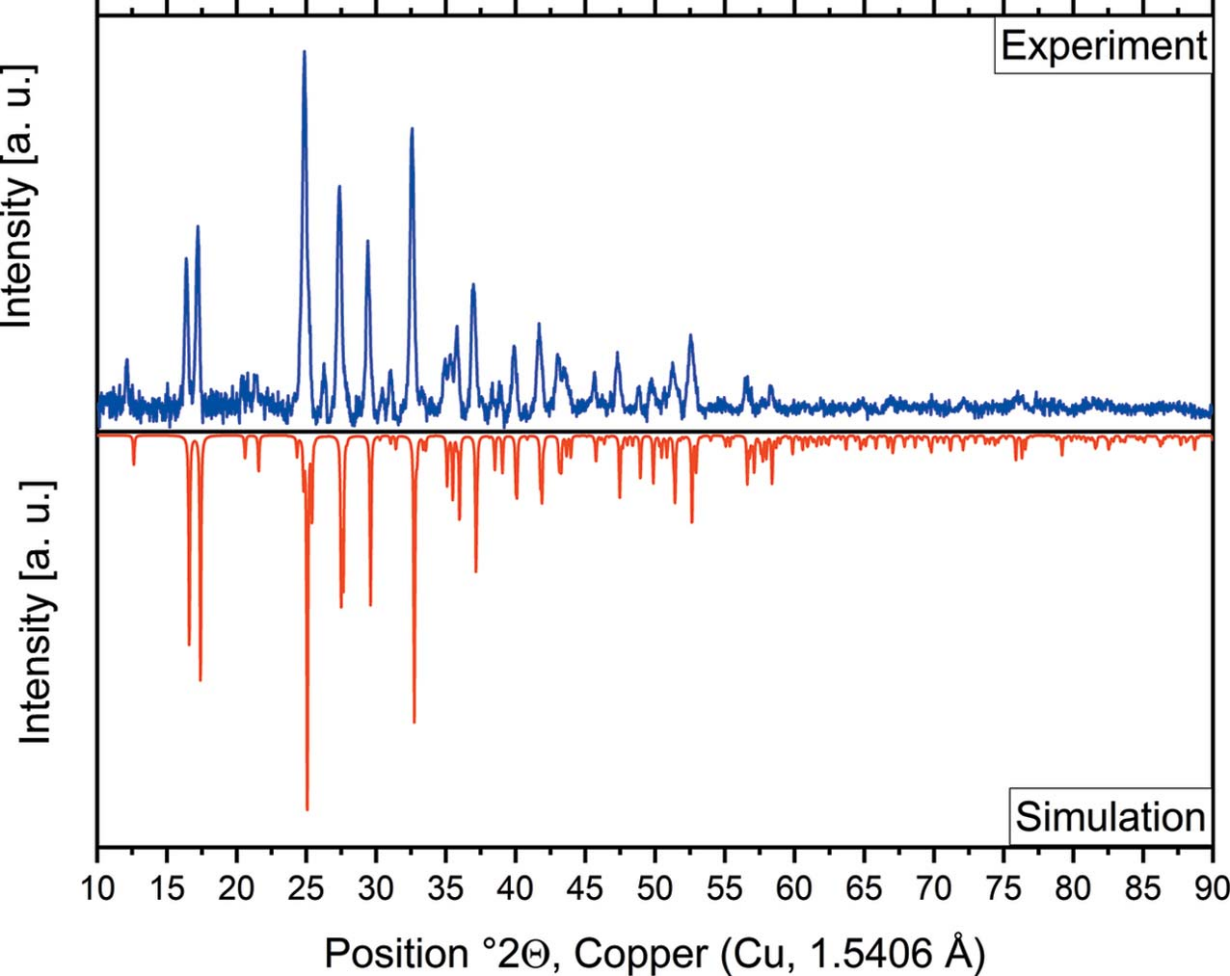
Experimental powder pattern of Na_2_[Hg_3_S_4_] on a PLA sample holder covered with Scotch Magic Tape (blue) compared with a simulated powder pattern from the crystal structure (red) (Klepp, 1992 ▸). Measured with Cu *K*α radiation (1.5406 Å) at 293 K.

Common PLA filament for 3D printing is not completely X-ray amorphous but shows very few distinct reflections. The positions of these reflections vary with storage time and printing temperature of the material. The specific background of the PLA sample holder should be determined by measuring an empty sample holder of the same batch of filament. Fig. 5 ▸ shows a comparison of the PXRD pattern of an empty PLA sample holder and two reference measurements of β-Na[FeO_2_] (Proskurnina *et al.*, 2020 ▸) on different sample holders: PLA and a commercially available silicon single-crystal disc. One of the most intense reflections of PLA is observed at 27° 2θ (Cu *K*α) next to a diffuse hump from 15 to 25° 2θ, which overlaps with the diffuse diffraction caused by the Scotch Magic Tape. Measurements of pure PLA show comparatively high intensities. When the PLA holder is covered with a sufficiently thick layer of powdered sample, the background contribution of the holder is nearly negligible (Fig. 5 ▸). Fig. 6 ▸ shows a measurement of Na_2_[Hg_3_S_4_] (Klepp, 1992 ▸). As Na_2_[Hg_3_S_4_] decomposes immediately when exposed to air, a comparison of the experimentally obtained diffractogram with the simulated powder pattern confirms that the setup using a 3D-printed PLA holder and Scotch Magic Tape successfully prevents the exposure to air during transport and the measurement time of 20 min.

## 4. Details on 3D printing

The 3D-printed pucks and the additional equipment can be designed with freely available 3D-modelling software. All the presented tools are made from PLA and printed by FDM with an Anycubic Mega S or Mega X 3D printer. PLA is a starch-based polymer, which can be degraded microbially under industrial compost conditions (Tokiwa *et al.*, 2009 ▸). Filament materials other than PLA may be employed but were not part of our investigations. PLA filament is convenient as it can be used with most commercially available FDM 3D printers at lower printing temperatures than, for example, acrylo­nitrile butadiene styrene.

### 4.1. Pucks and equipment for storage of single crystals

The 3D-printed pucks are custom-sized to fit into the sieves of the storage dewar. Depending on the size of the storage dewar, stacks with, for example, five pucks containing eight magnetic pins each can be stored simultaneously. For a smaller storage dewar, it could be three pucks with four pins each [Fig. 1 ▸(*a*) and Figs. S4(*a*) and 4(*b*)]. As PLA shrinks in liquid nitro­gen (77 K), it is crucial that the diameter of the cavity for the pin is 1.5–2 mm larger than the magnetic base to ensure that the pins do not get stuck. The material infill of the pucks needs to be ≥75%, to prevent buoyancy in the liquid nitro­gen which could result in the pucks tipping over and pins falling out. The space-holder pucks can be printed with a lower infill of approximately 50% and smaller diameter. The material cost of a large puck for eight samples is about 3.00 EUR (94 g per puck at 75% infill, 29.90 EUR per 1 kg of PLA filament). The pucks were used for about 1.5 years, enduring around 100 freeze–thaw cycles each, without any signs of cracks or damage. Owing to temperature-induced shrinkage and expansion, PLA is not recommended as a material for 3D-printed pins or magnetic bases. The crystal would move out of the beam as the pin and magnetic base are unevenly exposed to the nitro­gen cryostream at different orientations of the goniometer. 3D models of different pucks for cryogenic and ambient temperature storage, the puck dewar with a suitable lid (Cymon, 2012 ▸), the magnetic wand, and a tool to help remove stuck pins from the pucks (Fig. S2) can be found as .stl files in the SI.

### 4.2. PXRD sample holders

In order to avoid the scotch tape cover causing a de­align­ment of the 3D-printed sample holders in the holder ring, the sample holders are designed to be slightly smaller than the cavity of the sample-holder rings of the diffractometer [Fig. 4 ▸(*d*)]. The infill can be chosen to preference as long as the sample holder surface is smooth and flat. The material cost of a single 3D-printed sample holder is 0.03 EUR (1 g per sample holder, 29.90 EUR per 1 kg of PLA filament). 3D models for the two types of sample holders are provided as .stl files in the SI.

## 5. Summary and conclusions

A method to separate sample preparation from measurement of powder and single-crystal X-ray diffraction experiments is presented. For non-automated single-crystal X-ray diffractometers, the preparation and storage of suitable single crystals is a crucial aspect of an efficient use of the limited and valuable instrument time. Existing storage solutions are used occasionally but not as frequently as for automated setups. As 3D printers are widely available today, the application of 3D-printed equipment allows researchers to test if the separation of the crystal selection and mounting procedure from the actual measurement is suitable for their respective home source before investing in cost-intensive commercial solutions. The storage of selected crystals in liquid nitro­gen proves a suitable approach for air- and moisture-sensitive samples, providing precautions are followed to keep the liquid nitro­gen dry and reduce exposure to air during transfer to a minimum. Extremely moisture- or air-sensitive samples, as well as very temperature-sensitive compounds that need permanent cryogenic temperatures or undergo phase transition at low temperatures, can be stored in the individually adapted pucks. The PLA sample holders for PXRD experiments allow the preparation and storage of a large number of samples and provide an alternative for samples that may be corrosive towards silicon single crystals. The sample holders can be single use and save time and material which would otherwise be spent on cleaning. Both setups are accessible at low cost and are flexibly adaptable to individual laboratory needs.

## Supplementary Material

Zip archive containing the printable stl files. DOI: 10.1107/S160057672200293X/oc5019sup1.zip

Supporting information file. DOI: 10.1107/S160057672200293X/oc5019sup2.pdf

A video of the described procedure. DOI: 10.1107/S160057672200293X/oc5019sup3.mp4

## References

[bb1] Achilli, E., Minguzzi, A., Visibile, A., Locatelli, C., Vertova, A., Naldoni, A., Rondinini, S., Auricchio, F., Marconi, S., Fracchia, M. & Ghigna, P. (2016). *J. Synchrotron Rad.* **23**, 622–628. 10.1107/S1600577515024480 26917152

[bb2] Cymon (2012). *Brilliant Cut Diamond Cleaned Up*, https://www.thingiverse.com/thing:19137.

[bb3] Eck, B. van, Le, L. D., Issa, D. & Dye, J. L. (1982). *Inorg. Chem.* **21**, 1966–1970.

[bb4] Garman, E. F. (2014). *Science*, **343**, 1102–1108. 10.1126/science.1247829 24604194

[bb5] Goto, A., Hondoh, T. & Mae, S. (1990). *J. Chem. Phys.* **93**, 1412–1417.

[bb6] Huang, C.-Y., Meier, N., Caffrey, M., Wang, M. & Olieric, V. (2020). *J. Appl. Cryst.* **53**, 854–859. 10.1107/S1600576720002897 PMC7312129 32684901

[bb7] IVF Store (2021). Scematic Image Storage Dewar, https://us.ivfstore.com/products/high-capacity-liquid-nitrogen-storage?variant=12255315099725.

[bb8] Kitson, P. J., Marshall, R. J., Long, D., Forgan, R. S. & Cronin, L. (2014). *Angew. Chem. Int. Ed.* **53**, 12723–12728. 10.1002/anie.201402654 25079230

[bb9] Klepp, K. O. (1992). *J. Alloys Compd.* **182**, 281–288.

[bb10] Macdonald, N. P., Bunton, G. L., Park, A. Y., Breadmore, M. C. & Kilah, N. L. (2017). *Anal. Chem.* **89**, 4405–4408. 10.1021/acs.analchem.7b00443 28319372

[bb11] Miller, B. W., Moore, J. W., Barrett, H. H., Fryé, T., Adler, S., Sery, J. & Furenlid, L. R. (2011). *Nucl. Instrum. Methods Phys. Res. A*, **659**, 262–268. 10.1016/j.nima.2011.08.051 PMC3244175 22199414

[bb12] Olsson, A. & Rennie, A. R. (2016). *J. Appl. Cryst.* **49**, 696–699.

[bb13] Ott, H. & Stuerzer, T. (2016). *Absolute Structure Determination of Light Atom Compounds*. Bruker AXS GmbH, Karlsruhe, Germany.

[bb14] Papp, G., Rossi, C., Janocha, R., Sorez, C., Lopez-Marrero, M., Astruc, A., McCarthy, A., Belrhali, H., Bowler, M. W. & Cipriani, F. (2017). *Acta Cryst.* D**73**, 829–840. 10.1107/S2059798317013742 PMC5633908 28994412

[bb15] Proskurnina, N. V., Voronin, V. I., Shekhtman, G. S. & Kabanova, N. A. (2020). *Ionics*, **26**, 2917–2926.

[bb16] Tokiwa, Y., Calabia, B. P., Ugwu, C. U. & Aiba, S. (2009). *Int. J. Mol. Sci.* **10**, 3722–3742. 10.3390/ijms10093722 PMC2769161 19865515

