# Supplementary material for: 3D-printed equipment to decouple (powder) X-ray diffraction sample preparation and measurement
Source: J Appl Crystallogr. 2022 Apr 29;55(Pt 3):686–92. doi: 10.1107/S160057672200293X (PMC9172037; doi:10.1107/S160057672200293X)
Supplement: Supplementary file 2 [file j-55-00686-sup2.pdf]

JOURNAL OF
APPLIED
CRYSTALLOGRAPHY

Volume 55 (2022)

Supporting information for article:

3D-printed equipment to decouple (P)XRD sample preparation and measurement

Friederike Fuß, Max Rieckert, Simon Steinhauer, Moritz Liesegang and Günther Thiele

S1. Details on the creation of the 3D models

3D-models of pucks, the dewar, the magnetic wand and the pin removal tool were made with the software *Fusion 360®* by *Autodesk* and edited with *Microsoft 3D builder*. The lid for the dewar and the PXRD sample holders were made with *Microsoft 3D builder*, for the lid a slice from a diamond model from thingiverse.com (Cymon, 14.03.2012) was used. The PLA filament was purchased at 3dk-Berlin. All equipment was printed with a layer thickness of 0.1 mm and a print speed of 60 mm/s.

S1.1. Additional equipment: Dewar, magnetic wand, pin-removal tool for pins stuck in the puck, room temperature pin holder for Schlenk tubes

The dewar to keep the pucks submerged in liquid nitrogen while transferring the pins to the diffractometer, is assembled from three 3D-printed parts. Additionally, commercially available single component polyurethane (PU) foam is required. The parts are glued together with a suitable household glue and the hollow space inside it is filled with PU foam. The nozzle of the foam can is inserted into the holes at the top of the dewar. PU foam expands a lot, thus if too much foam is added it can cause the single parts to separate again. Any excess foam can be cut off with a knife after hardening. The direct exposure of PU foam to liquid nitrogen should be avoided as this will cause the foam to implode. The lid of the dewar has a slit for the wire of the last puck in the stack (Figure S1).

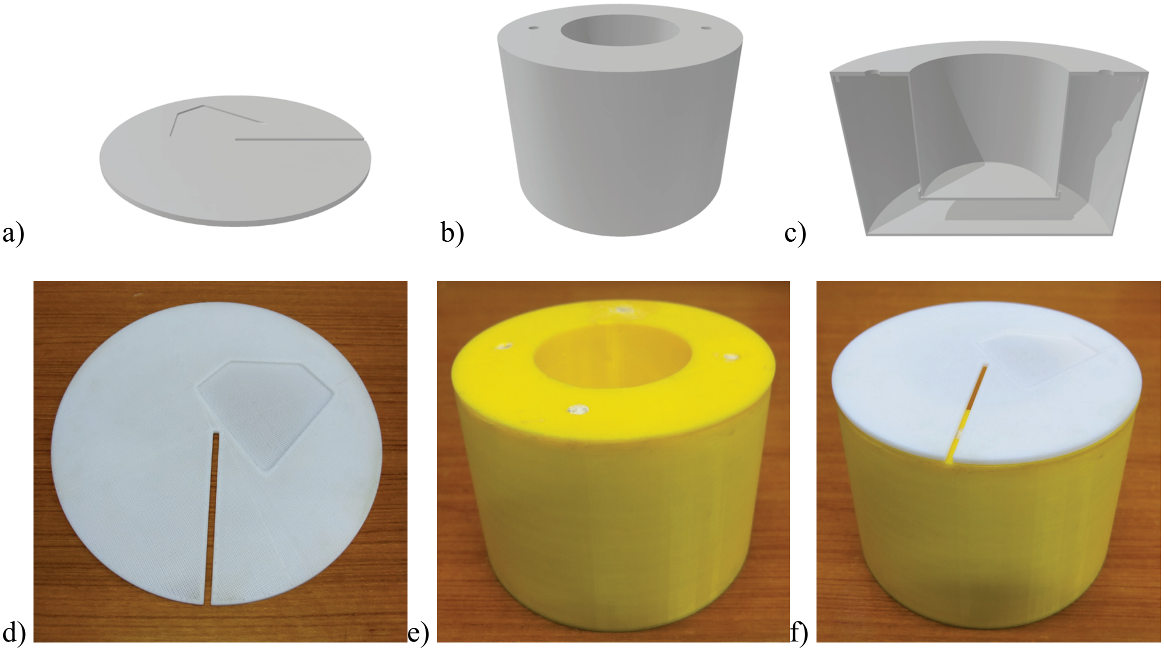

a) b) c) d) e) f)

Figure S1 Schematic depictions of a) Lid of the dewar for a single puck. b) Fully assembled dewar, holes on the top are for inserting PU foam. c) Cross-section of the dewar exposing the hollow spaces

to be filled with PU foam. Photos of d) Lid of the dewar for a single puck. e) Fully assembled dewar filled with PU foam. f) Dewar with lid.

The magnetic wand is composed of a 3D-printed handle and a disc-shaped permanent magnet attached with glue. Two models for magnetic wands are attached in this document, mainly differing in the size of the head and the cavity for the permanent magnet.
If a pin gets stuck within the puck, a pin removal tool is placed in the cavity from below to help release it (Figure S2).

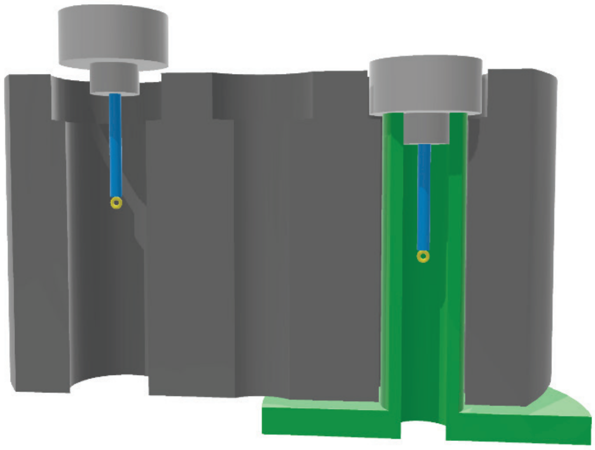

a) b)

Figure S2 a) Schematic depiction of a pin removal tool (green) to help release pins stuck in the puck. b) Photo of the pin removal tool.

For samples which undergo phase transitions at low temperature a pin holder was designed which can be inserted into a common Schlenk tube to store the crystal at ambient temperature protected from air and moisture (Figure S3). The selected crystal in the pin holder is inserted into the argon flushed Schlenk tube and the tube then closed. For longer Schlenk tubes a space-holder can be inserted below the pin holder, to allow easy access to the pin. If several crystals are chosen, the pucks can be threaded onto a wire. The respective .stl files are provided below.

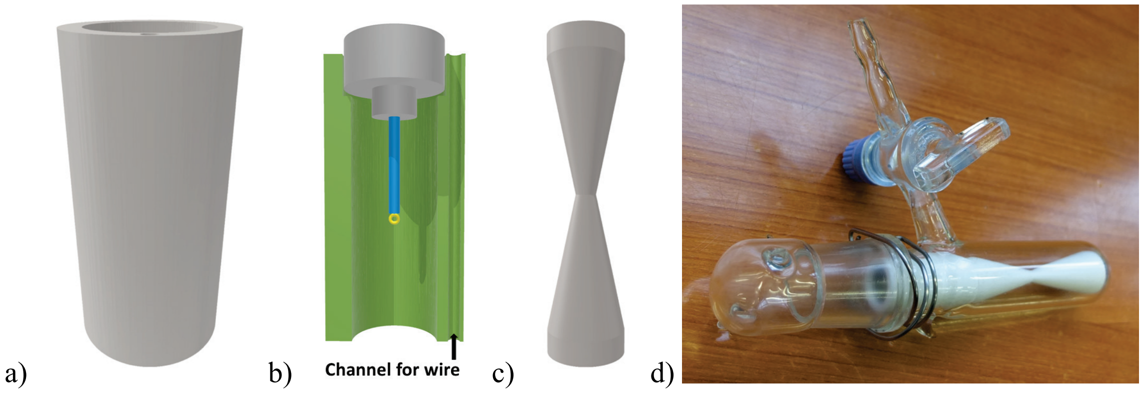

Figure S3 Schematic depictions of a) Puck for a single pin for storage of temperature-sensitive samples in a Schlenk tube. b) Cross section of the puck with a pin inside. c) Space-holder for the single puck. Photo of d) A puck for a single pin with a space-holder inside a Schlenk tube.

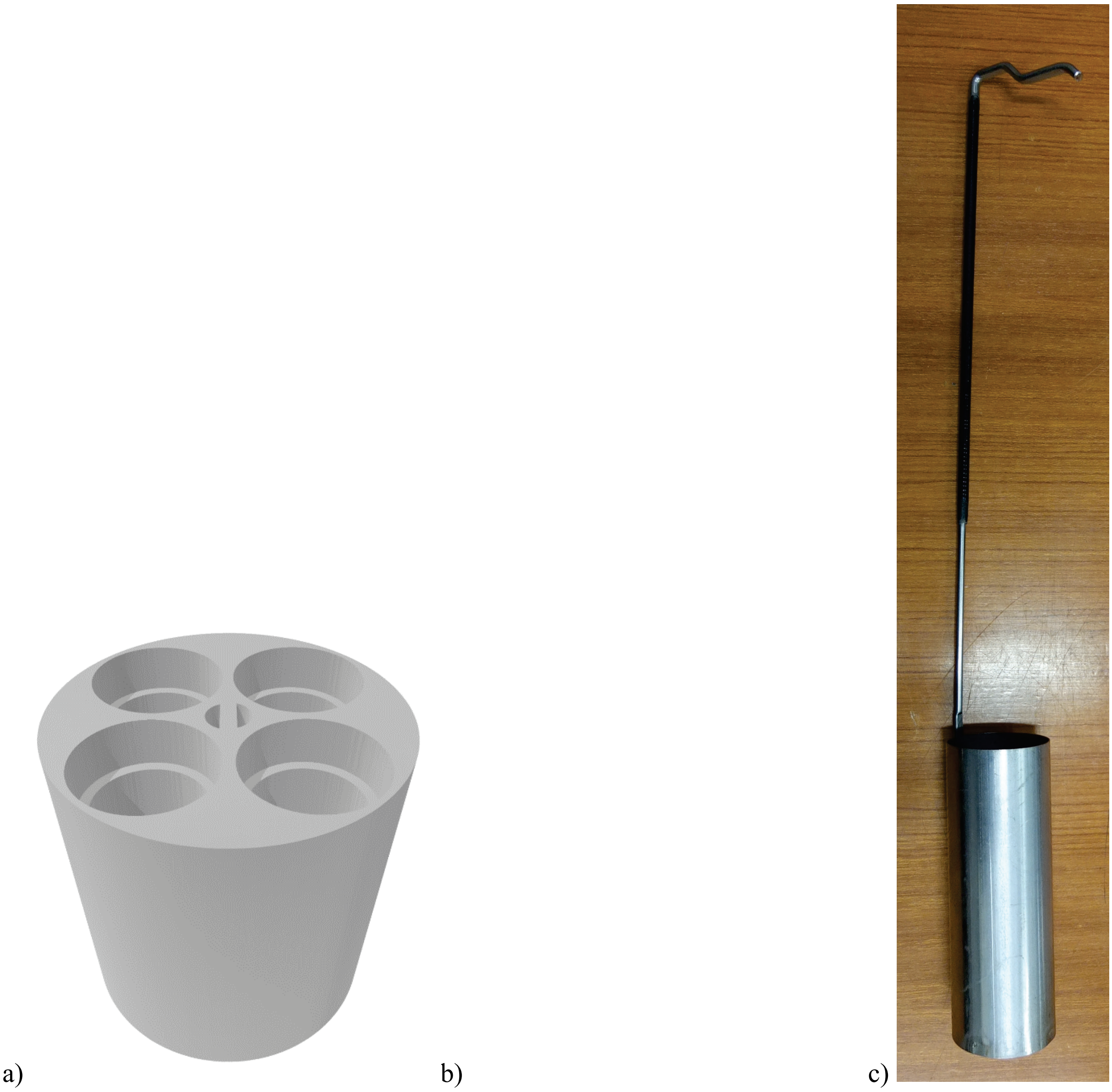

Figure S4 a) Schematic depiction of a small puck for storage of four pins. Photos of b) A small puck for the storage of four pins. c) A sieve for small pucks.

S2. Measurement details

S2.1. Single crystal X-Ray experiments

Crystal images were recorded at 100 K in a nitrogen gas flow prior to the measurement on a *Bruker D8 Venture* single crystal diffractometer with a Photon 100-CMOS-detector, goniometer with kappa-geometry and a low temperature unit.

S2.2. Powder X-Ray diffraction experiments

Powder diffraction data was recorded on a *Panalytical Empyrean* diffractometer with Cu K_{α}-radiation (1.5406 Å) and a *PIXcel* 1D detector with 255x255 pixels at a PHD-level of 45-70% to supress X-ray

fluorescence of iron. The sample holders were covered in *Scotch Magic™ Tape*. Empty PLA holders and references with *β*-Na[FeO_2] were measured on a rotating stage, at a step size of 0.026° 2Θ and scan speed of about 5° 2Θ per minute. The slight shift of reflexes to lower 2Θ angles due to a dislocation of the sample in the beam is systematic and can be considered as such. Diffraction data of Na_2[Hg_3S_4] was recorded on a rotating stage at a step size of 0.026° 2Θ and scan speed of 4° 2Θ per minute. Background correction for the diffractogram of Na_2[Hg_3S_4] was carried out with *X'pert High Score* by *Panalytical*.

S3. List of attached .stl-files

PXRD-sample-holder-hollow

PXRD-sample-holder-flat

Cryopuck-big

Cryopuck-small

MagneticWandv1

MagneticWandv2

Pin-removal-tool

Dewar1 – upper part of the dewar

Dewar2 – outer part of the dewar

Dewar3 – bottom of inner cone of the dewar

Lid-dewar (The diamond shape in the lid was made using a model "Brilliant Cut Diamond Cleaned Up" from (Cymon, 14.03.2012) and is licensed under a creative commons attribute and share alike license. "This work is licensed under the Creative Commons Attribution-ShareAlike 3.0 Unported License. To view a copy of this license, visit http://creativecommons.org/licenses/by-sa/3.0/ or send a letter to Creative Commons, PO Box 1866, Mountain View, CA 94042, USA."

Cryopuck-big_closed_bottom

Single-puck

Space-holder-single-puck

S4. Limitations of compatibility with automated setups

The pucks and all the additional setup were designed similar to devices for automated setups. Due to the material choice and the standard deviations the presented tools are not compatible with automated setups.

S5. Additional photos and a video of equipment and setups

In the following photos of the equipment are shown (Figure S5 and S6). Additionally a video showing the operation of the equipment for single crystal storage is provided with the .stl files.

a) 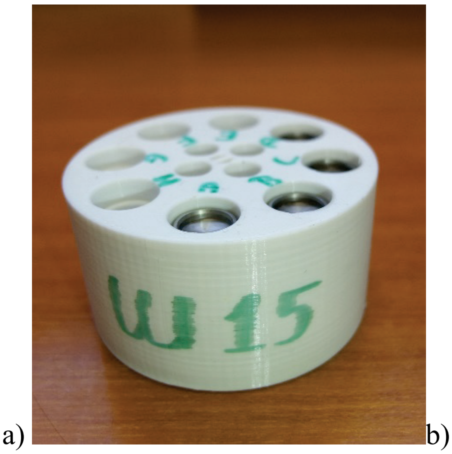

b)

Figure S5 Photos of a) A puck filled with pins. The puck identifier on the side and the pin position labels are shown on top. b) Magnetic wand.

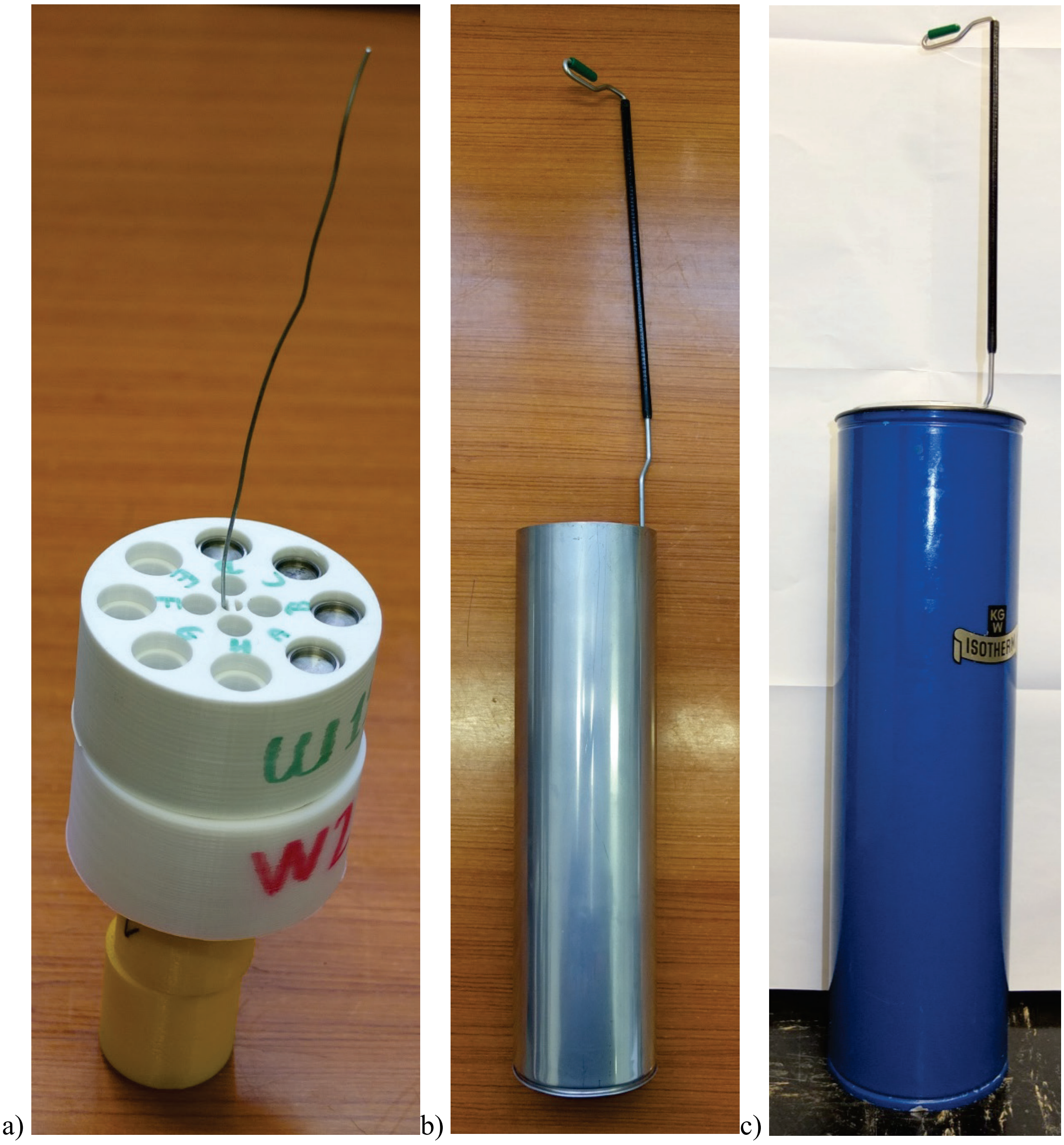

a) b) c)

Figure S6 Photos of a) Stack of pucks, with two yellow space-holder pucks below. b) Sieve from the cryogenic sample storage dewar, where the stack of pucks is located inside. c) Sieve submerged in dewar, as would be used at the microscope, the Styrofoam lid used at the microscope is not shown here.
